# Supplementary material for: Intracellular accumulation of free cholesterol in macrophages triggers a PARP1 response to DNA damage and PARP1 impairs lipopolysaccharide-induced inflammatory response
Source: PLoS One. 2025 Mar 5;20(3):e0318267. doi: 10.1371/journal.pone.0318267 (PMC11882048; doi:10.1371/journal.pone.0318267)
Supplement: S1 Fig — Real-time OCR measurements were performed using a Seahorse analyzer and PMφs with or without oxLDL accumulation (±oxLDL) and LPS stimulation. OCR measurements were normalized to one reading cycle prior to LPS injection (indicated by an arrow). The mean ± SEM is plotted (n = 4). Significant differences between corresponding time points in –oxLDL versus + oxLDL groups were not detected using a one-way ANOVA and a Bonferroni post hoc test. (PDF) [file pone.0318267.s001.pdf]

S1 Fig

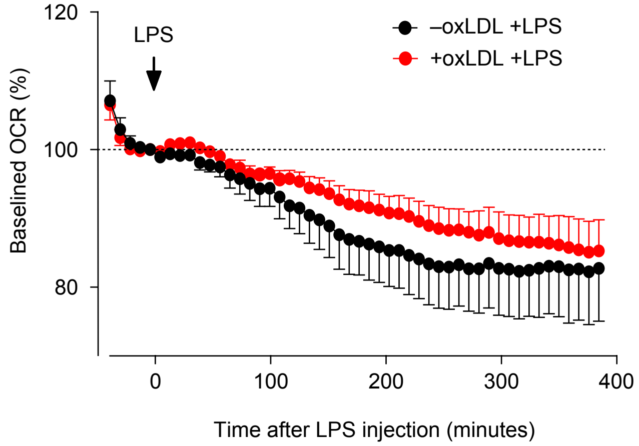

Characterizing the effect of oxLDL loading on the metabolic profile of PMφs after LPS stimulation.

Real-time OCR measurements were performed using a Seahorse analyzer and PMφs with or without oxLDL accumulation (±oxLDL) and LPS stimulation. OCR measurements were normalized to one reading cycle prior to LPS injection (indicated by an arrow). The mean ± SEM is plotted (n = 4). Significant differences between corresponding time points in –oxLDL versus +oxLDL groups were not detected using a one-way ANOVA and a Bonferroni post hoc test.
