# Supplementary material for: Intracellular accumulation of free cholesterol in macrophages triggers a PARP1 response to DNA damage and PARP1 impairs lipopolysaccharide-induced inflammatory response
Source: PLoS One. 2025 Mar 5;20(3):e0318267. doi: 10.1371/journal.pone.0318267 (PMC11882048; doi:10.1371/journal.pone.0318267)
Supplement: S2 Fig — (A, B) Quantification of NAD + consumption in PMφs. FK-866, a NAMPT inhibitor, and rucaparib, a PARP1 inhibitor, were added every 15 minutes for up to 1 h. For each group, the data are normalized to NAD + values just prior to the addition of FK-866 and rucaparib (0 min time point, assigned a value of 1). (A) PMφs with or without accumulated cholesterol without LPS stimulation (n = 3). (B) PMφs with or without accumulated cholesterol and 3 h after LPS stimulation (n = 3). (C) ABCA1 protein expression detected by immunoblotting PMφs with or without free cholesterol loading (n = 3). Rucaparib or DMSO control was added 1 h prior to culturing cells with or without cholesterol for 24 h. The ABCA1 values are normalized to corresponding actin and the –Rupacarib and –Chol group (assigned a value of 1). The mean ± SEM is plotted in all graphs. Significant differences were determined by a two-way ANOVA and a Bonferroni post hoc test ( * P < 0.05). (PDF) [file pone.0318267.s002.pdf]

S2 Fig

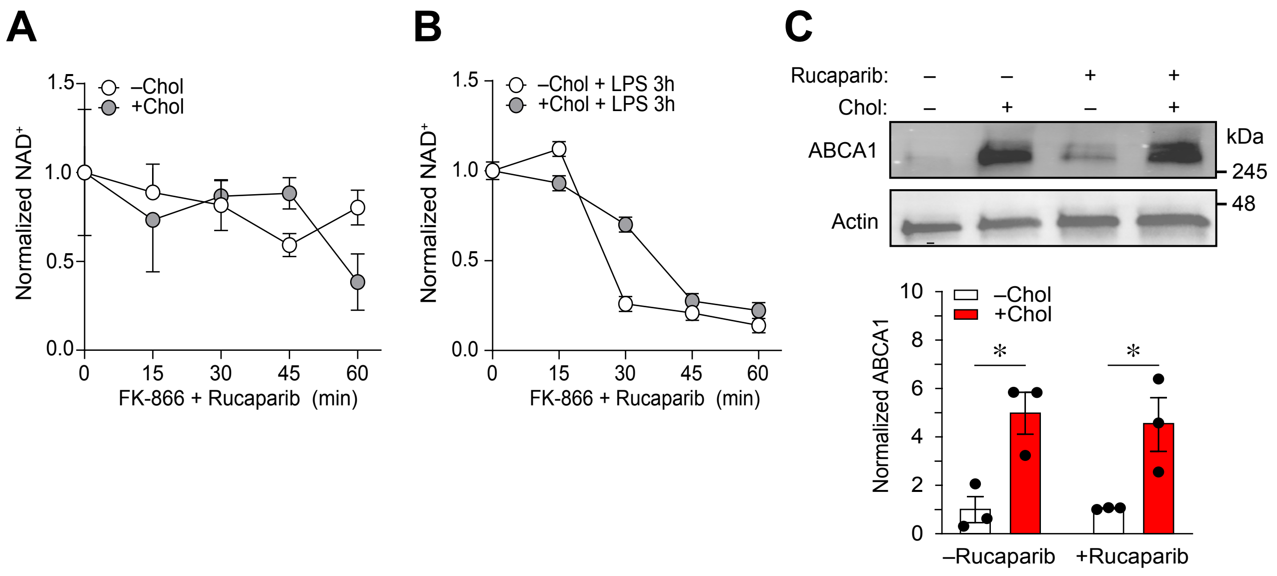

Characterizing the effect of rucaparib on cholesterol-loaded PMφs.

(A, B) Quantification of NAD+ consumption in PMφs. FK-866, a NAMPT inhibitor, and rucaparib, a PARP1 inhibitor, were added every 15 minutes for up to 1 h. For each group, the data are normalized to NAD^+ values just prior to the initial addition of FK-866 and rucaparib (0 min time point, assigned a value of 1). (A) PMφs with or without accumulated cholesterol without LPS stimulation (n = 3). **(B)** PMφs with or without accumulated cholesterol and 3 h after LPS stimulation (n = 3). **(C)** ABCA1 protein expression detected by immunoblotting PMφs with or without free cholesterol loading (n=3). Rucaparib or DMSO control was added 1 h prior to culturing cells with or without cholesterol for 24 h. The ABCA1 values are normalized to corresponding actin and the –Rupacarib and –Chol group (assigned a value of 1). The mean ± SEM is plotted in all graphs. Significant differences were determined by a two-way ANOVA and a Bonferroni post hoc test (* $P < 0.05$).
