# Supplementary material for: Intracellular accumulation of free cholesterol in macrophages triggers a PARP1 response to DNA damage and PARP1 impairs lipopolysaccharide-induced inflammatory response
Source: PLoS One. 2025 Mar 5;20(3):e0318267. doi: 10.1371/journal.pone.0318267 (PMC11882048; doi:10.1371/journal.pone.0318267)
Supplement: S3 Fig — (A, B) AG14361, a PARP1 inhibitor, was added 1 h prior to culturing PMφs with or without free cholesterol (Chol, A, n = 3-5) or oxLDL (B, n = 3–5). Subsequently PMφs were stimulated with LPS for 6 h, as is shown in a schematic above each graph. Inflammatory gene expression was measured by q PCR. The mean ± SEM is plotted (n = 3). Significant differences were determined using an unpaired Student’s t test ( * P < 0.05, ** P < 0.01, *** P < 0.001, **** P < 0.0001). (PDF) [file pone.0318267.s003.pdf]

S3 Fig

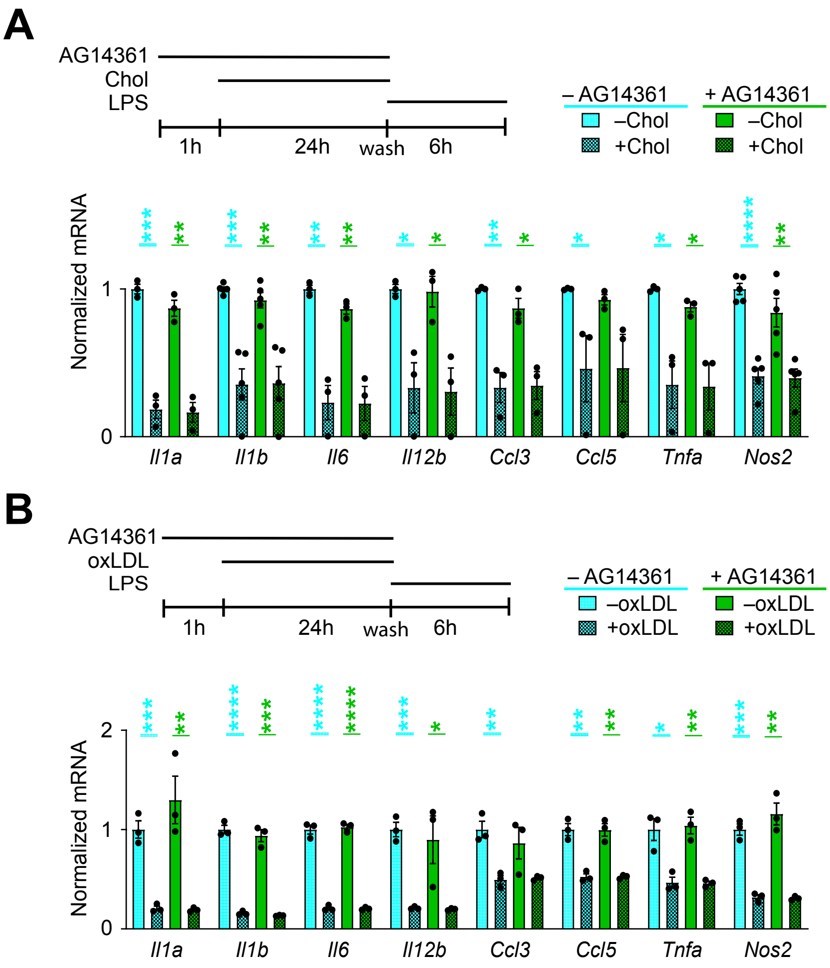

Inhibition of PARP1 enzymatic activity does not rescue the suppression of LPS-induced inflammatory gene expression in lipid-loaded PMφs.

(A, B) AG14361 was added 1 h prior to culturing PMφs with or without free cholesterol (Chol, A, n = 3-5) or oxLDL (B, n = 3-5). Subsequently PMφs were stimulated with LPS for 6 h, as is shown in a schematic above each graph. Inflammatory gene expression was measured by qPCR. The mean ± SEM is plotted. Significant differences were determined using an unpaired Student's t test (* $P < 0.05$, ** $P < 0.01$, *** $P < 0.001$, **** $P < 0.0001$).
