# Supplementary material for: Intracellular accumulation of free cholesterol in macrophages triggers a PARP1 response to DNA damage and PARP1 impairs lipopolysaccharide-induced inflammatory response
Source: PLoS One. 2025 Mar 5;20(3):e0318267. doi: 10.1371/journal.pone.0318267 (PMC11882048; doi:10.1371/journal.pone.0318267)

Figure 1D p53

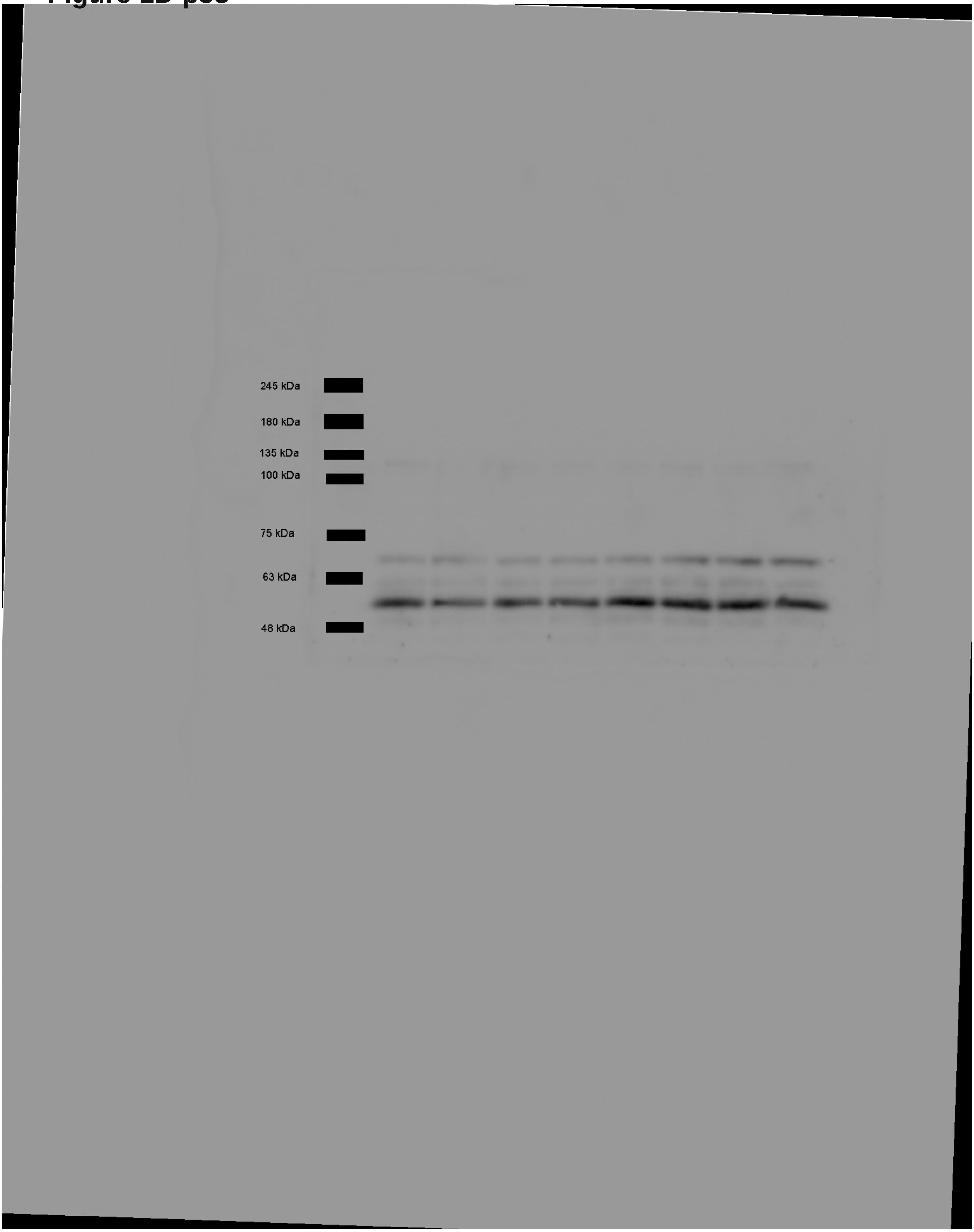

Figure 1D Lamin A/C

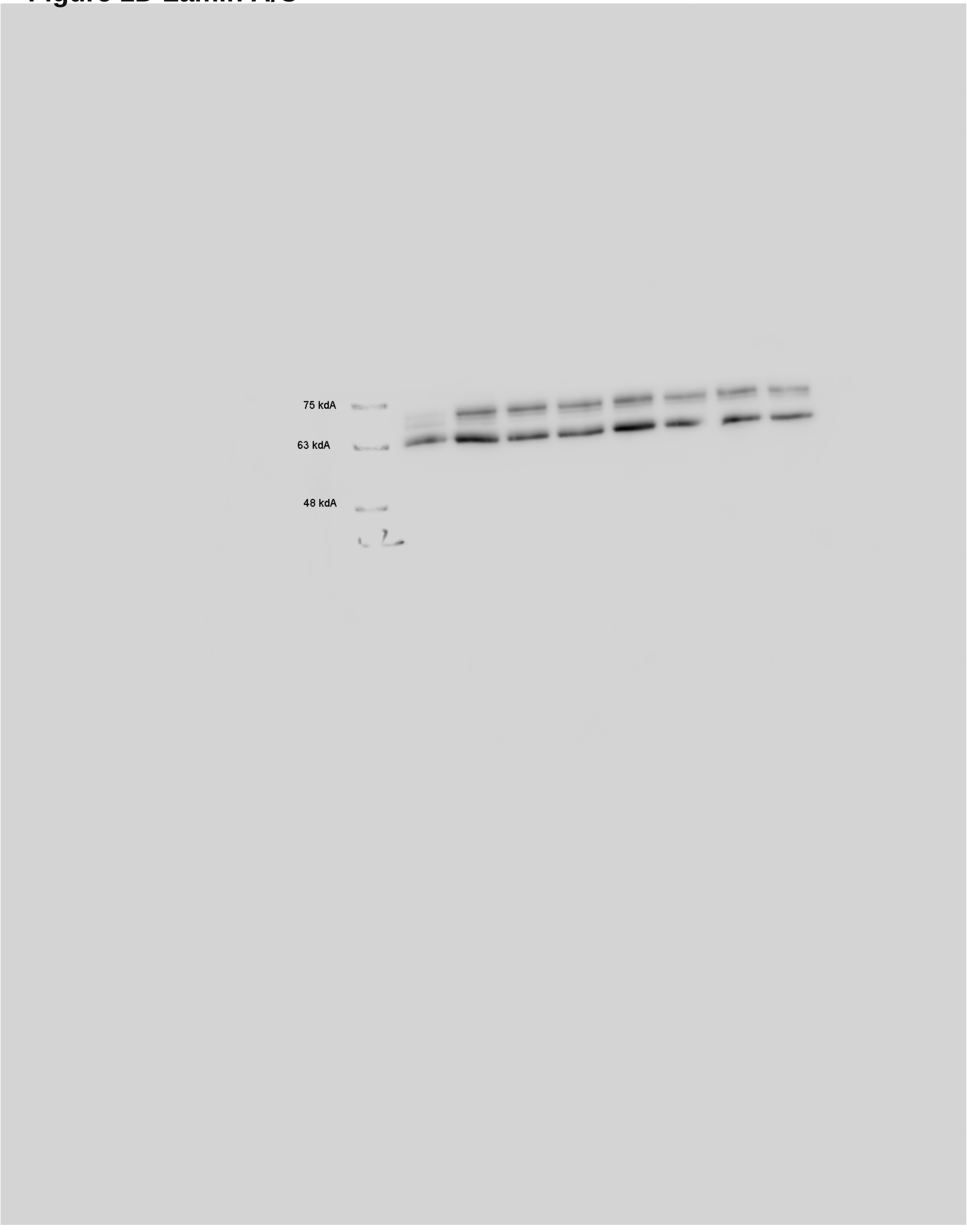

Figure 2A PAR

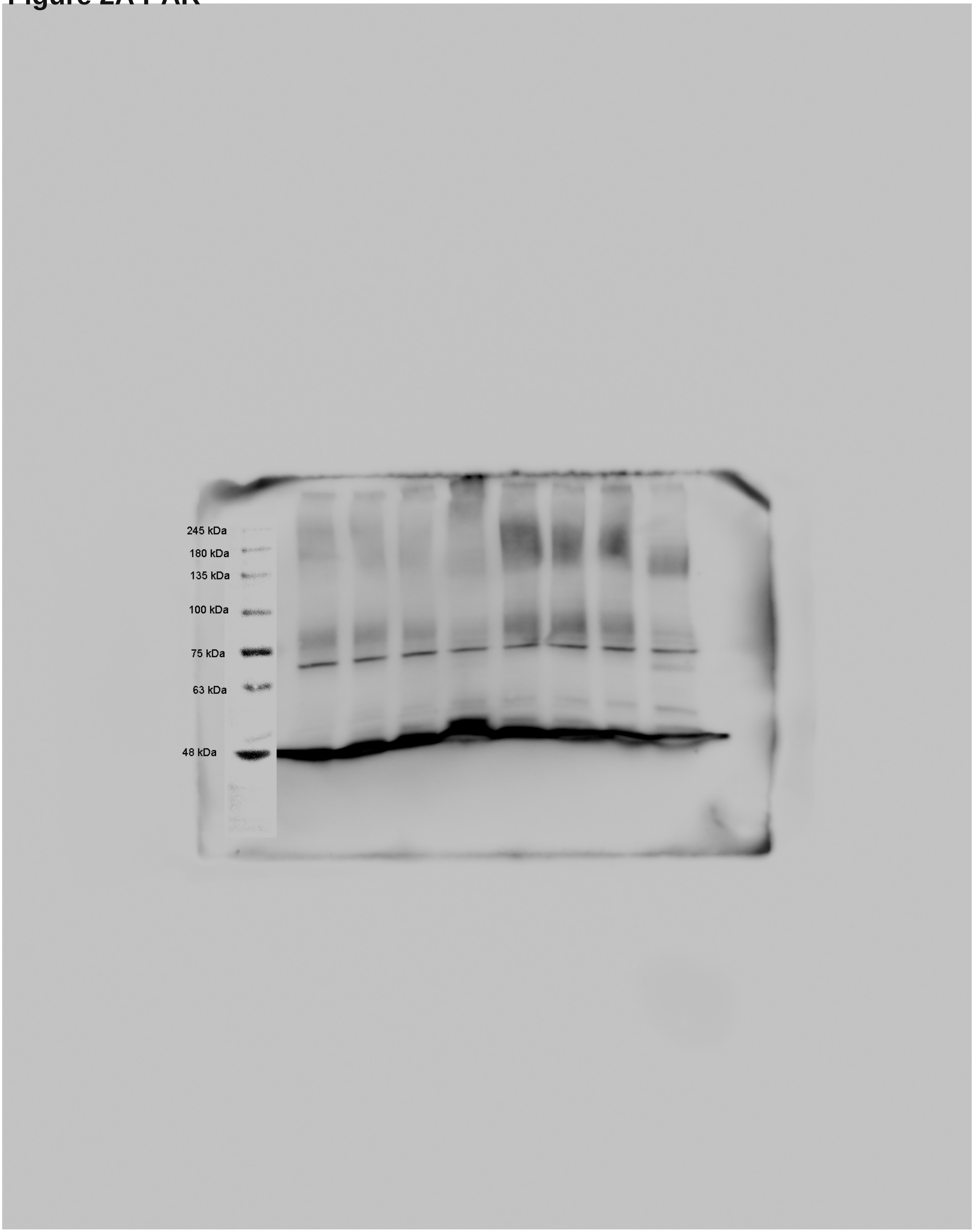

Figure 2A Actin

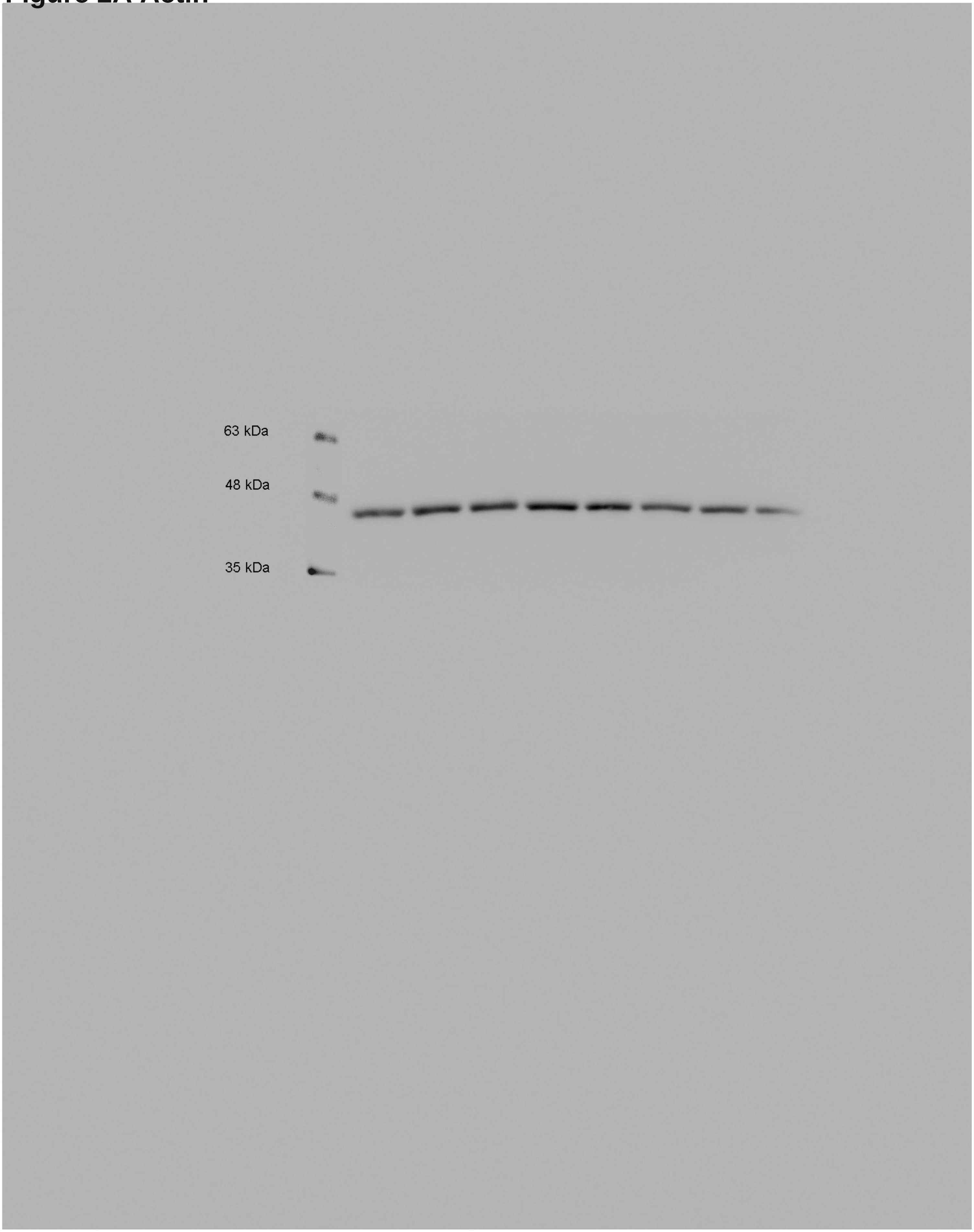

Figure 2A PARP1

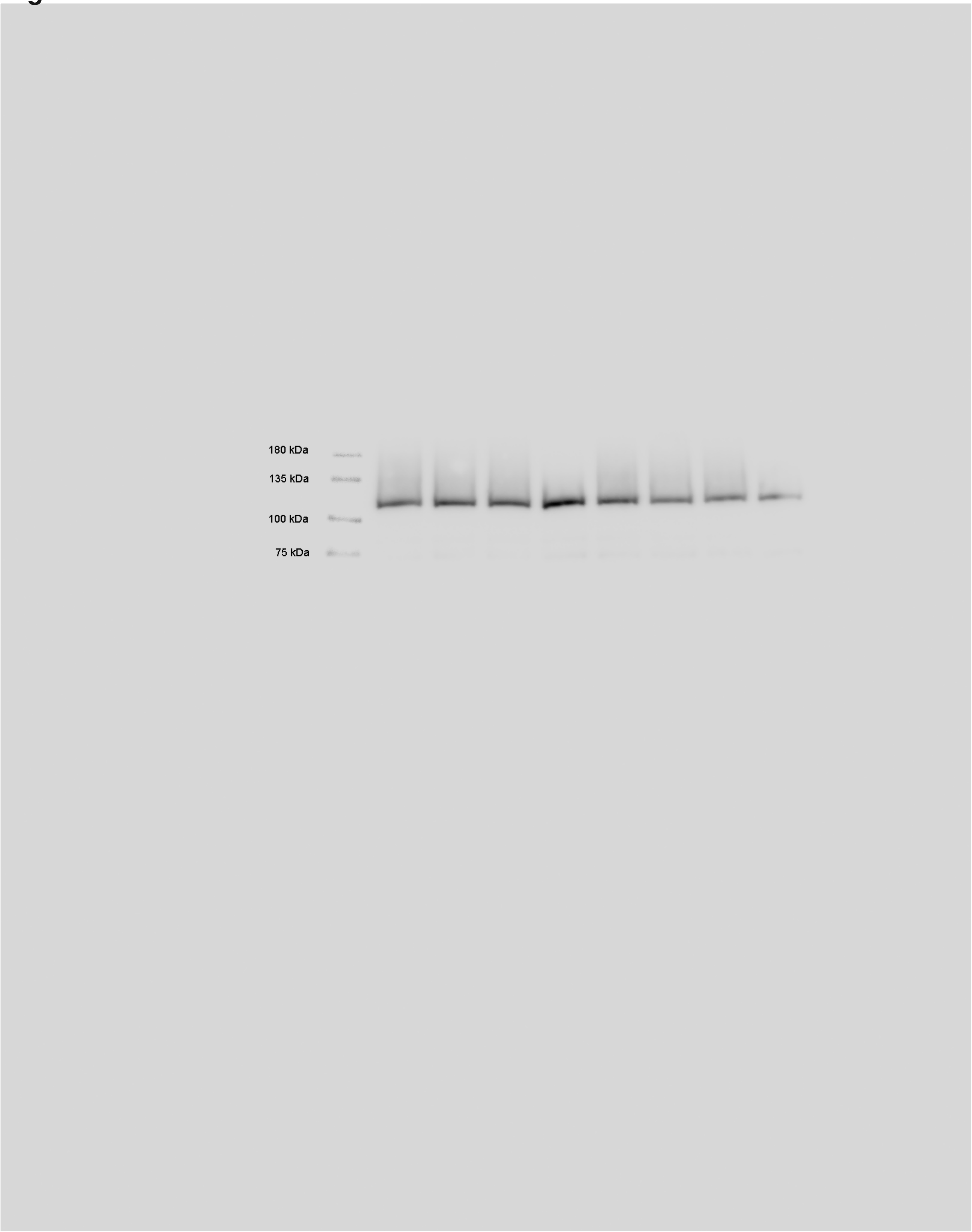

Figure 2B actin

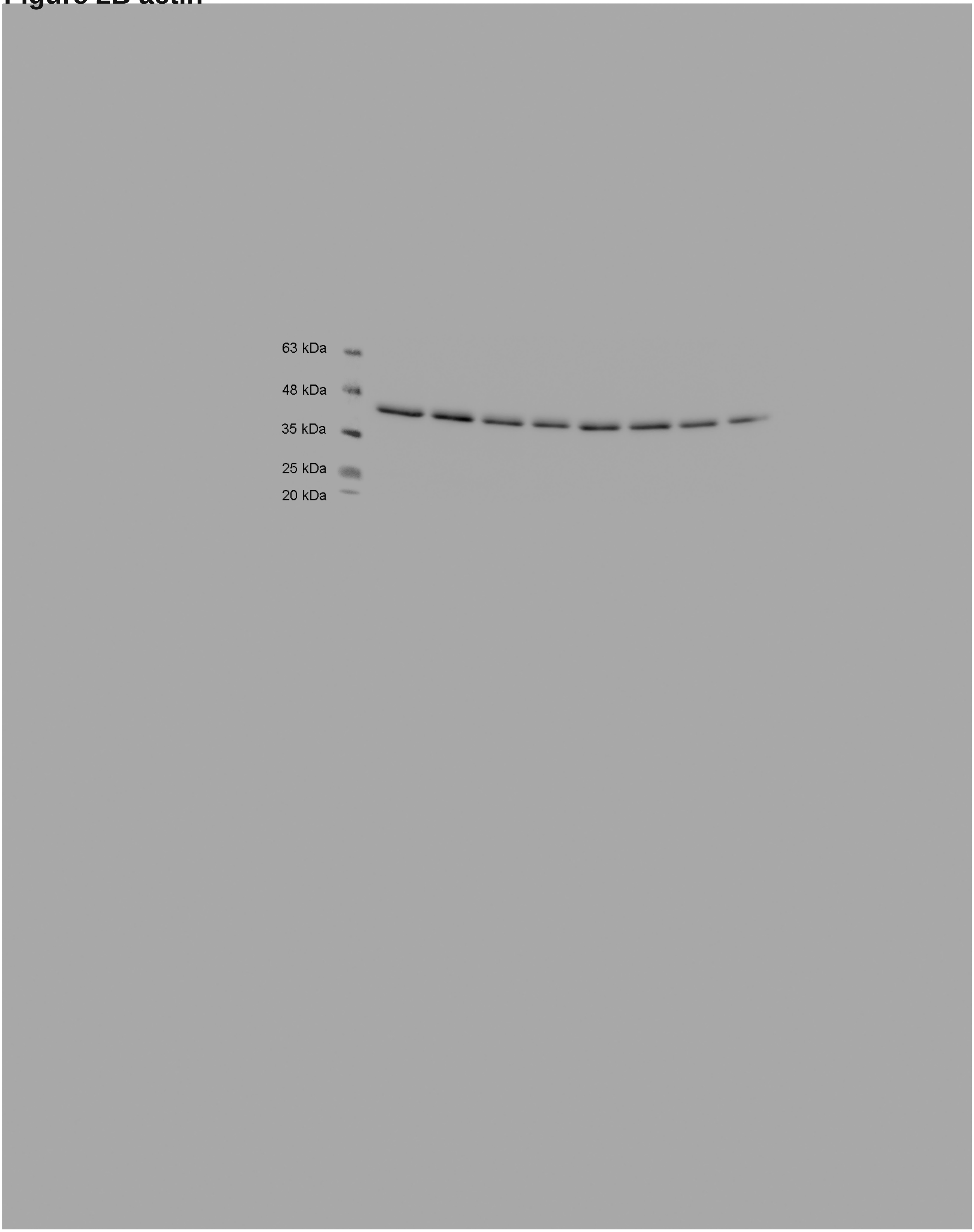

Figure 2B PAR

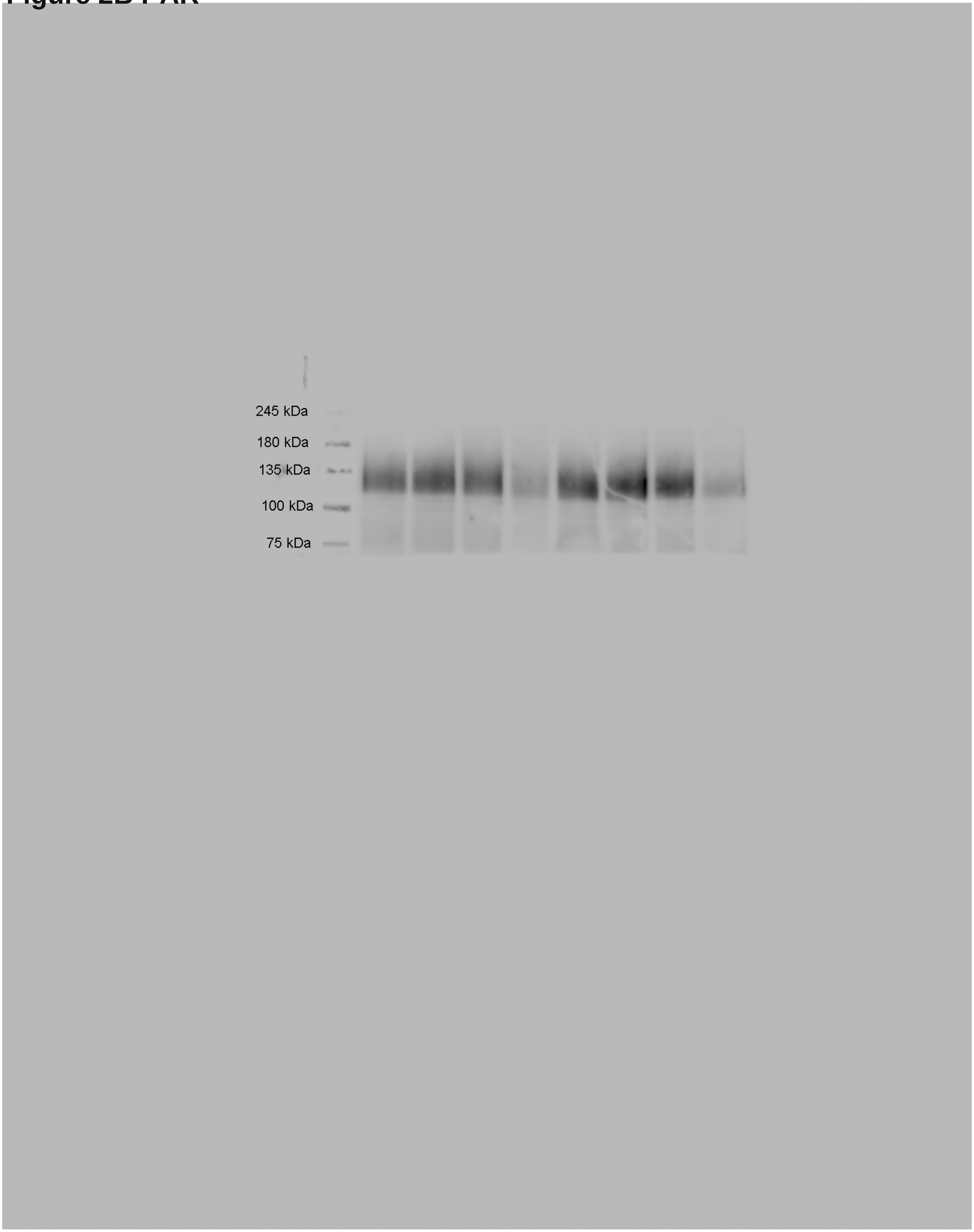

Figure 2B PARP1

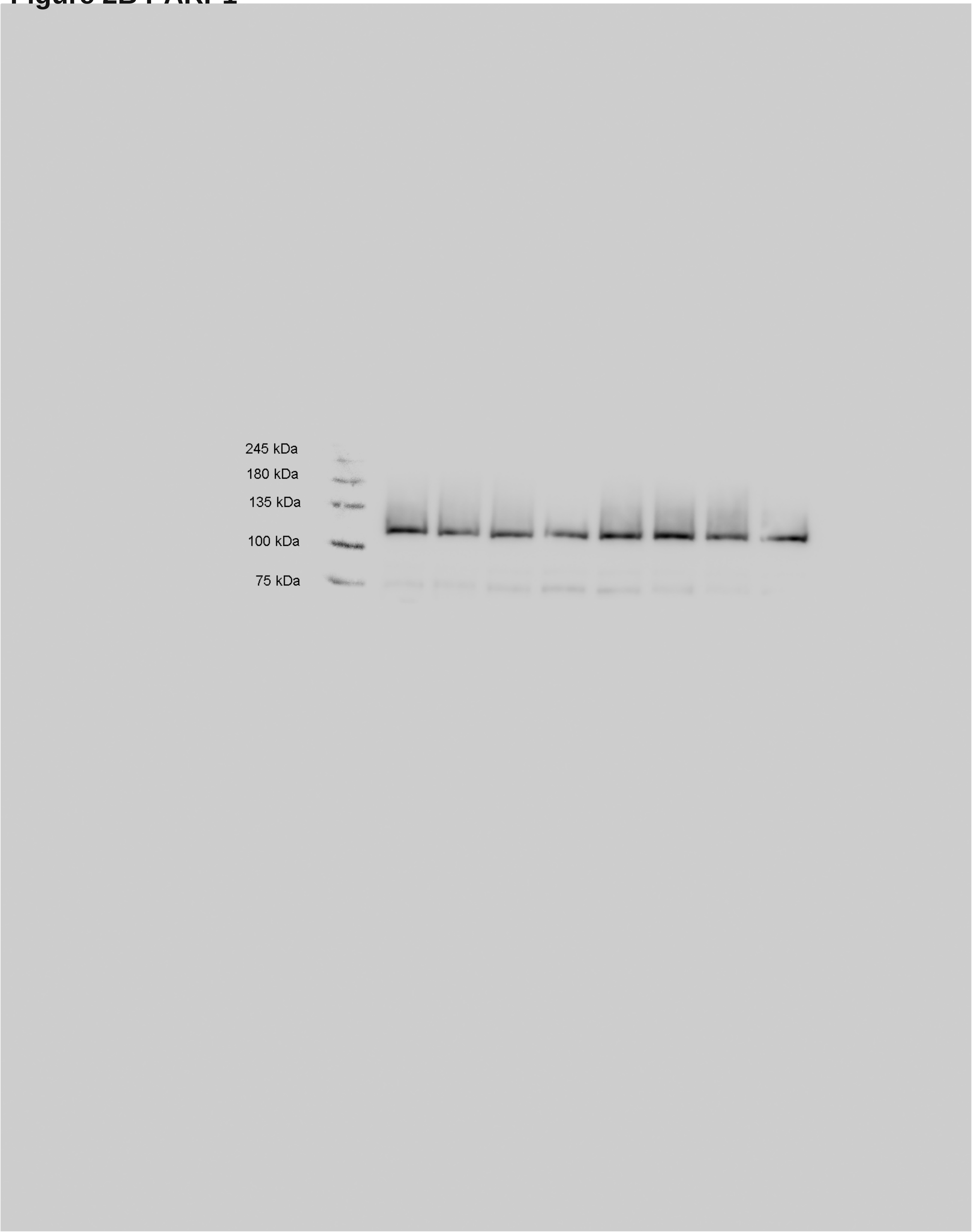

Figure 2C PAR

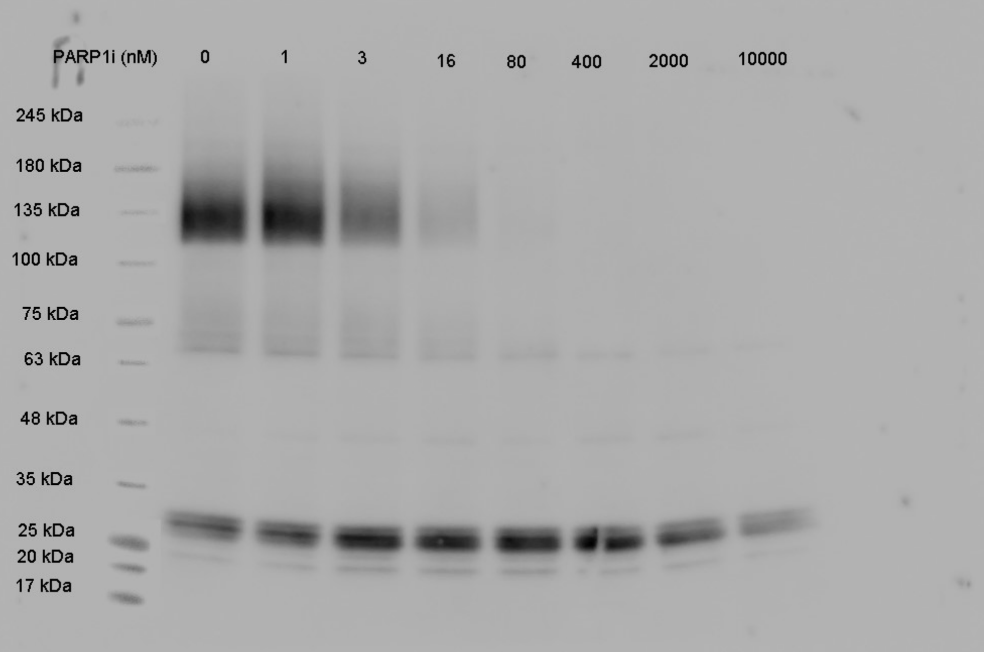

Figure 2C Actin

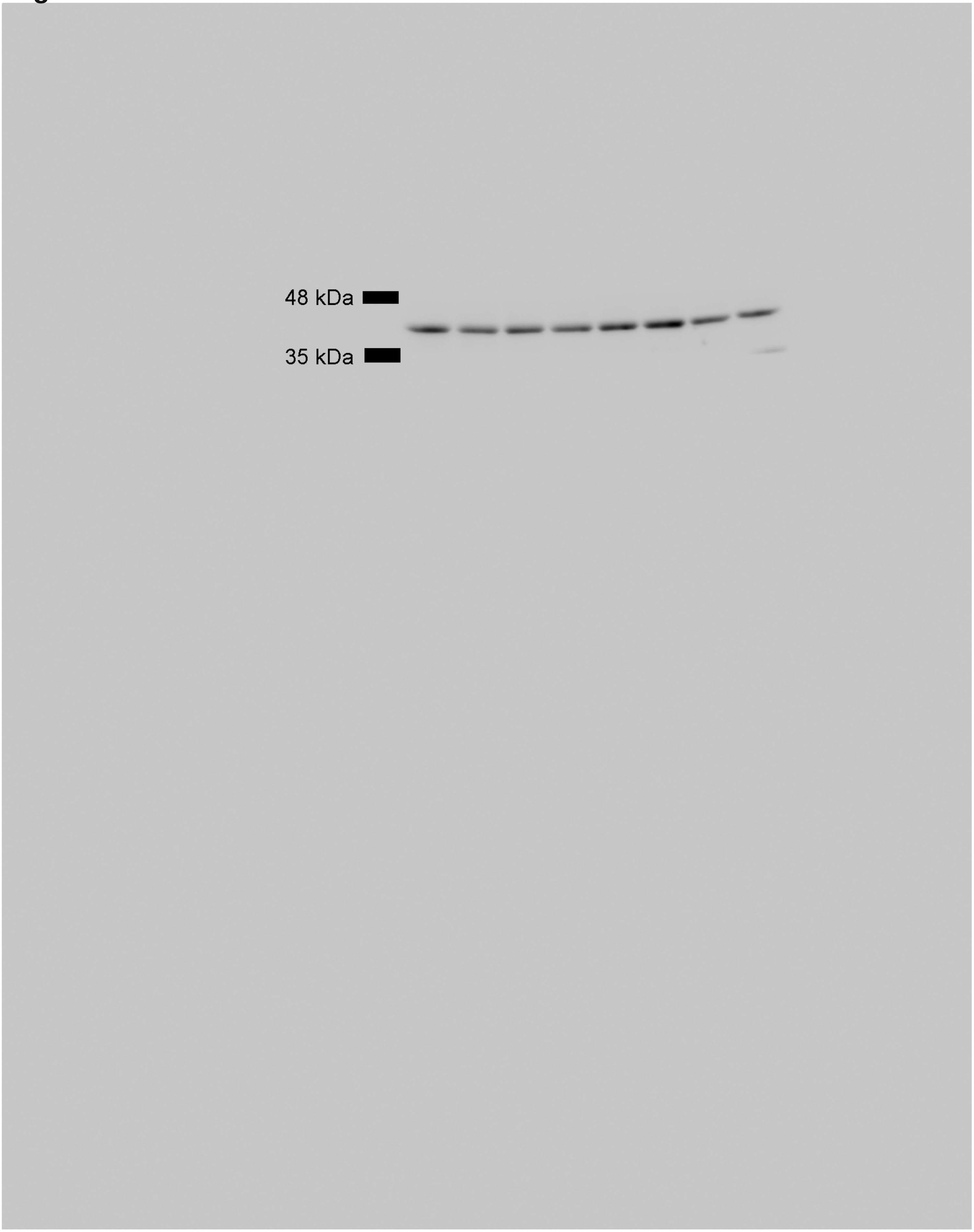

Figure 2D y-H2A.X

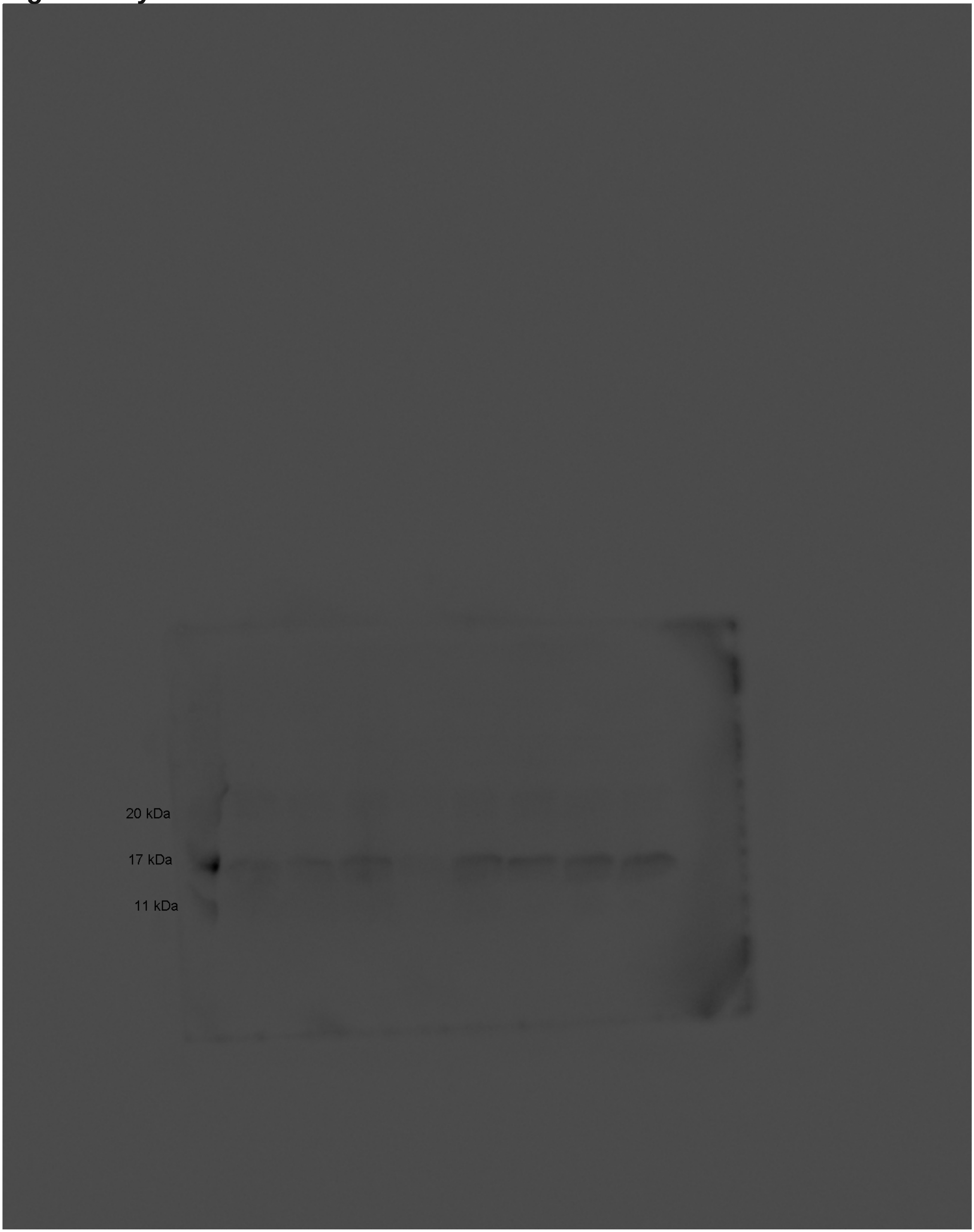

Figure 2D H2A.X

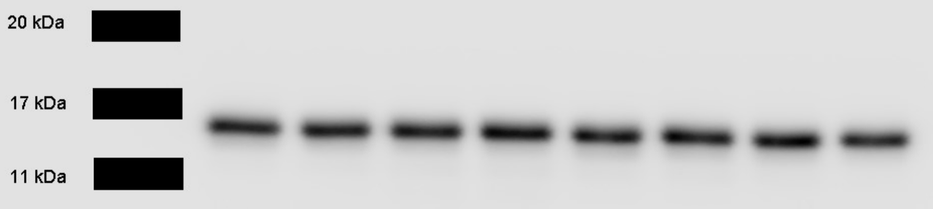

Figure 4C PAR

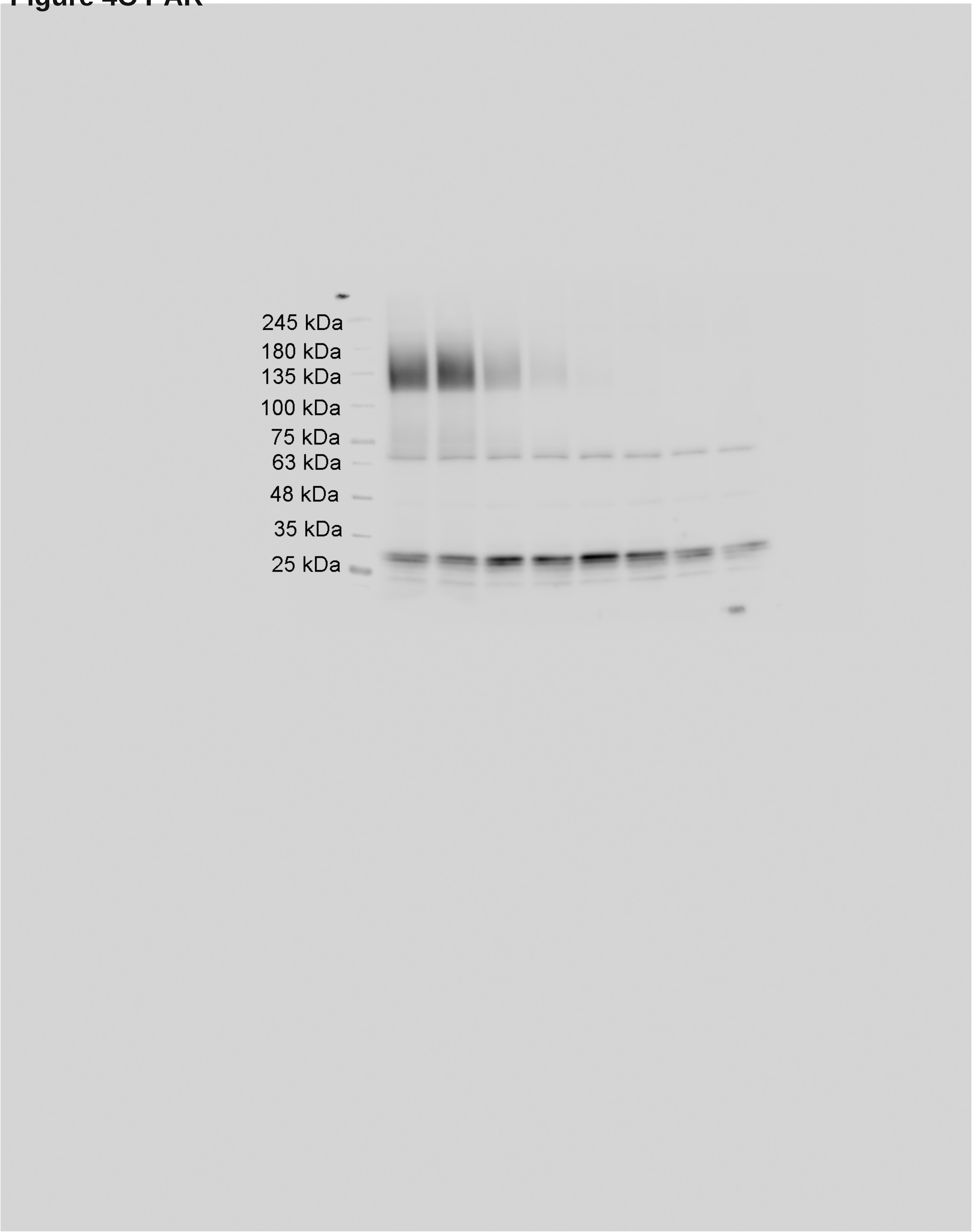

Figure 4C actin

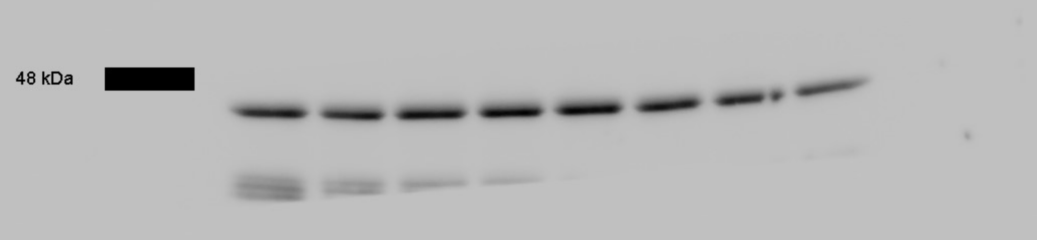

Figure 6B PARP1

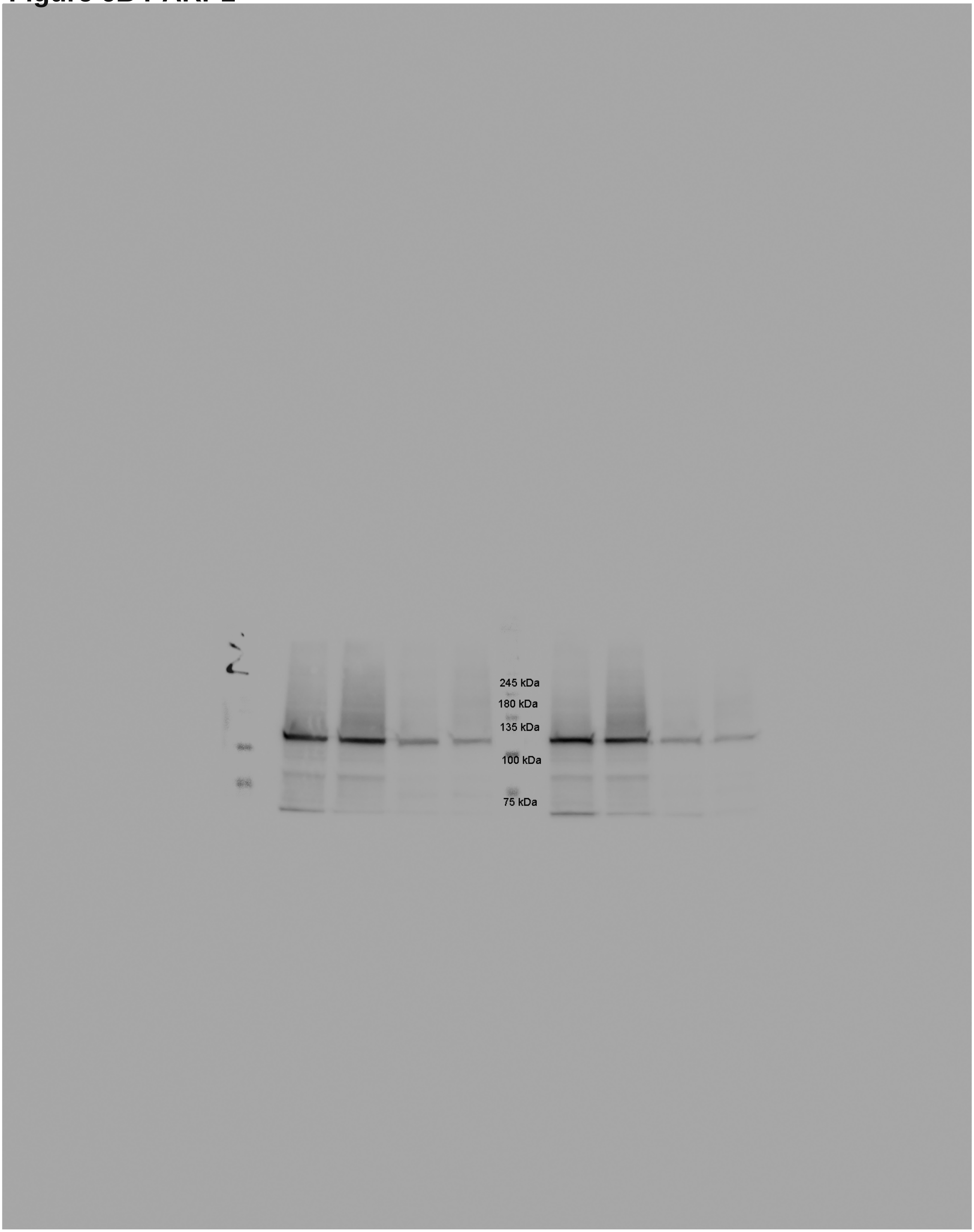

Figure 6B actin

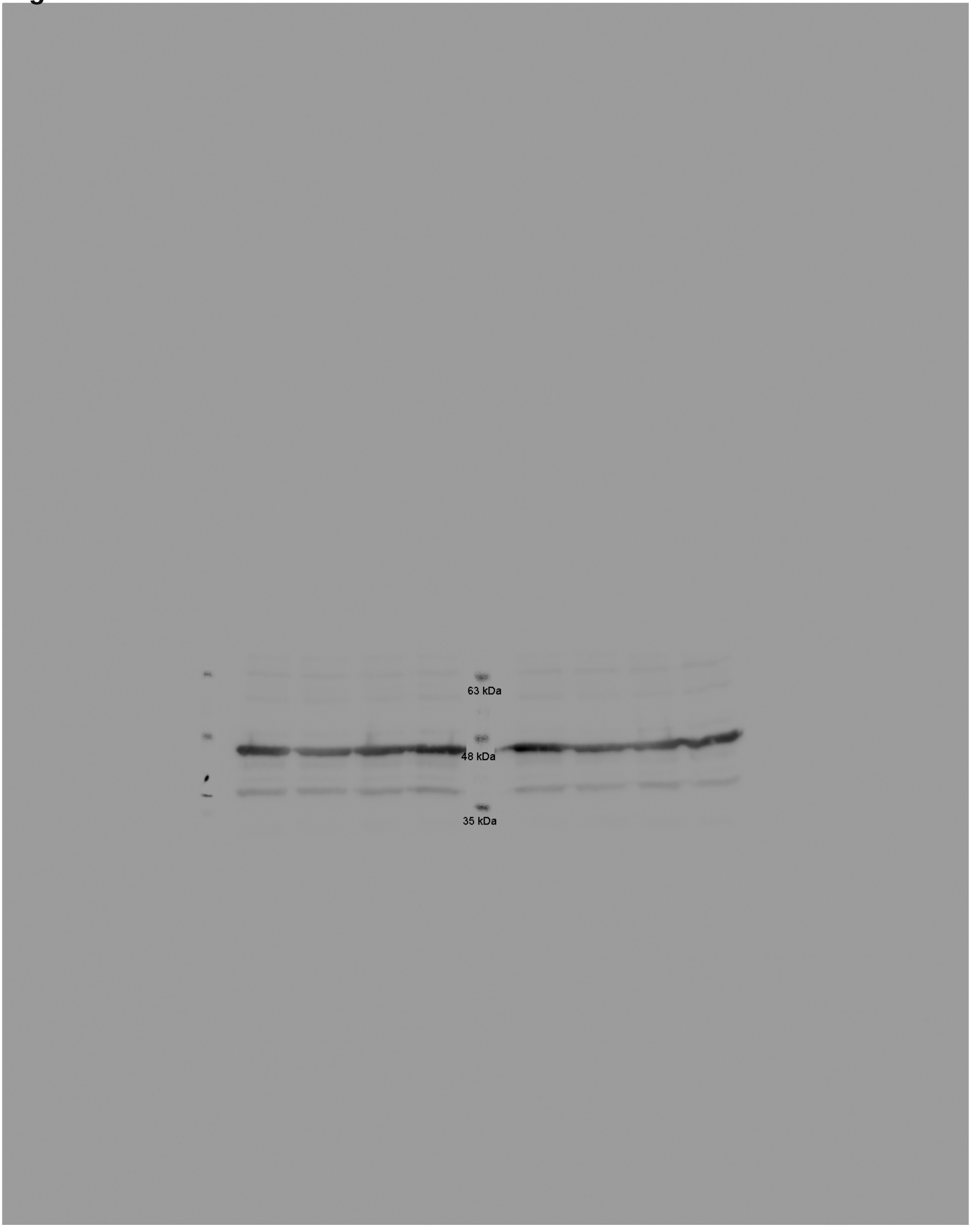

Supplement: S4 Fig — (PDF) [file pone.0318267.s004.pdf]
